# Mass ivermectin treatment for Onchocerciasis: Lack of evidence for collateral impact on transmission of *Wuchereria bancrofti *in areas of co-endemicity

**DOI:** 10.1186/1475-2883-4-6

**Published:** 2005-07-15

**Authors:** FO Richards, A Eigege, D Pam, A Kal, A Lenhart, JOA Oneyka, MY Jinadu, ES Miri

**Affiliations:** 1 The Carter Center, One Copenhill, Atlanta GA 30307, USA; 2 Department of Zoology, University of Jos, Jos, Plateau State, Nigeria; 3 Room 913, Phase II, Federal Secretariat, Federal Ministry of Health, Lagos, Nigeria

## Abstract

There has long been interest in determining if mass ivermectin administration for onchocerciasis has 'unknowingly' interrupted lymphatic filariasis (LF) transmission where the endemicity of the two diseases' overlaps. We studied 11 communities in central Nigeria entomologically for LF by performing mosquito dissections on *Anopheline *LF vectors. Six of the communities studied were located within an onchocerciasis treatment zone, and five were located outside of that zone. Communities inside the treatment zone had been offered ivermectin treatment for two-five years, with a mean coverage of 81% of the eligible population (range 58–95%). We found 4.9% of mosquitoes were infected with any larval stage of *W. bancrofti *in the head or thorax in 362 dissections in the untreated villages compared to 4.7% infected in 549 dissections in the ivermectin treated villages (Mantel-Haenszel ChiSquare 0.02, P = 0.9). We concluded that ivermectin annual therapy for onchocerciasis has not interrupted transmission of Wuchereria bancrofti (the causative agent of LF in Nigeria).

## Findings

Ivermectin is an effective microfilaricidal oral medication that is being distributed in mass drug administration programmes for two filarial diseases, onchocerciasis [1] and lymphatic filariasis (LF) [2,3]. Both onchocerciasis and LF are vector borne, with onchocerciasis transmitted by *Simulium *black flies, and LF by *Anopheline *mosquitoes in rural Africa. Merck and Co. donates ivermectin (Mectizan^®^) to global control programmes for both these parasitic diseases, although annual ivermectin in combination with albendazole (donated by GlaxoSmithKline) is recommended by WHO for the treatment of LF in Africa, because of the presumed synergy [4,5], although this remains in debate [6].

Of the two initiatives, the oldest is that for onchocerciasis and ivermectin has been distributed in annual ivermectin monotherapy (150 micrograms/kg) programmes in Africa for over 16 years [1]. There has long been interest in determining if such ivermectin distribution for onchocerciasis has 'unknowingly' interrupted LF transmission where the endemicity of the two diseases' overlaps [7]. We had occasion to address this question in central Nigeria in 1999 while conducting *Anopheline *entomological sampling for LF in and outside of onchocerciasis programme zones.

The study was performed in Plateau and Nasarawa States, Nigeria, as part of an integrated onchocerciasis, schistosomiasis, and lymphatic filariaisis programme described by Hopkins et al. [8]. Twelve of the 30 local government areas (LGA) in these two states are onchocerciasis treatment zones and have been offered annual ivermectin monotherapy since 1993. LF mapping in 1998 designated all 30 LGA for combined ivermectin and albendazole mass treatment for LF. Prior to launching the larger LF treatment programme, we sought (in 1999) entomology sentinel sites for a longitudinal study of treatment impact on LF transmission [9]. To identify villages with high baseline infection rates, our team captured and dissected resting *Anopheles gambiae sl *and *An. funestus *in randomly selected households in 11 villages, 5 of which were outside of the onchocerciasis ivermectin treatment zone, and 6 were inside the treatment zone. Treatment coverage for those six ivermectin treatment villages during the years 1995–1999 ranged from 58–95% of the eligible population (Table 1) with a mean of 81%.

**Table:** Ivermectin treatment coverage of the eligible population (1995–1999) and LF antigenemia (1999) among male residents in five ivermectin treated villages, with 1999 LF antigenemia in one untreated village (Gwamlar)

**Village**	**Angwan Lemu**	**Apanda**	**Bakin-Kogi**	**Lankan**	**Mungkohot**	**Gwamlar**
**Ivermectin rounds**	2	2	2	5	5	-
**Mean coverage (range)**	91.4% (91–92)	66.9% (58–76)	85.3% (82–89)	85.9% (65–95)	77% (73–80)	-
**1999 coverage**	92%	58%	89%	90%	80%	-
**% LF antigenemia in males (n)**	40% (30)	43% (30)	27% (30)	47% (30)	47% (30)	58% (50)

After obtaining permission from local village chiefs and residents of the selected household, trained collectors used aspirators and torches to capture indoor resting Anopheline mosquitoes; 75% of these were Anopheles gambiae sl, the remainder were An.funestus. The mosquitoes, most of which were blood fed, were immediately transferred to screened paper cups and kept alive in an ice chest containing wet towels until dissected later that same day. At that time the mosquitoes were killed, placed on a glass slide, separated into head, abdomen and thorax, teased apart in normal saline, and examined under a binocular microscope. Infection rates were based on the finding of any larval stage of *W. bancrofti *in head or thorax. Microfilaria in the abdomen where not considered in the infection rate calculations.

LF antigenemia testing occurred on a separate occasion using the rapid ICT card test [10] (AMRAD Corporation Ltd., North South Wales, Australia). The test was performed as described by Eigege [11] on finger stick blood samples of 30 randomly selected adult male residents from five of the six treated villages and in 50 individuals in one of the six untreated villages (Gwamlar).

We found that the untreated village of Gwamlar, had both the highest mosquito infection rate (20%) and the highest antigenemia rate (58%). However, no statistically significant entomological differences could be demonstrated between the villages in treated and untreated zones (Figure 1): 4.9% of mosquitoes were infected in 362 dissections in the untreated villages compared to 4.7% infected in 549 dissections in the ivermectin treated villages (Mantel-Haenszel ChiSquare 0.02, P = 0.9).

In contrast LF antigenemia (Table 1) was less common among the 150 adult residents examined in the ivermectin treated villages (mean 41%, village range 27–47%) compared to the untreated village of Gwamlar, having the forementioned 58% antigenemia prevalence (ChiSquare 4.5, P = 0.03).

We conclude therefore, that ivermectin monotherapy for onchocerciasis has not been sufficient to interrupt transmission of LF in central Nigeria. Among treated villages, mosquito infection rates in treated and untreated areas were statistically equivalent, and antigenemia rates in treated villages were unacceptably high (although lower than those in Gwamlar). Mosquito infection rates were indeed highest in the two villages (Lankan and Mungkohot) with the longest treatment history (5 years) with adequate coverage. Our conclusion is in support of the findings of Kyelem et al., [7] who, working in Burkina Faso, demonstrated that ivermectin monotherapy given twice per year for onchocerciasis reduced but did not interrupt LF transmission there.

## Competing interests

The author(s) declare that they have no competing interests.

## Authors' contributions

Drs. Richards, Eigege, Jinadu, and Miri and Professor Oneyka were involved in the design, supervision, analysis and preparation of the manuscript. Mr. Pam and Mr. Kal supervised the fieldwork and performed the dissections, under the field supervision of Professor Oneyka. Ms Lenhart played a major role in data analysis.

## References

[B1] Richards FO, Boatin B, Sauerbrey M, Sékétéli A (2001). Control of Onchocerciasis Today: Status and Challenges. Trends Parasitol.

[B2] Brown KR, Ricci FM, Ottesen EA (2000). Ivermectin: effectiveness in lymphatic filariasis. Parasitology.

[B3] Molyneux DH, Zagaria N (2002). Lymphatic filariasis elimination: progress in global programme development. Annals Trop Med Parasitol.

[B4] Addiss DG, Beach MJ, Streit TG, Lutwick S, LeConte FH, Lafontant JG, Hightower AW, Lammie PJ (1997). Randomised placebo-controlled comparison of ivermectin and albendazole alone and in combination for Wuchereria bancrofti microfilaraemia in Haitian children. Lancet.

[B5] Ottesen EA, Ismail MM, Horton J (1999). The role of albendazole in programmes to eliminate lymphatic filariasis. Parasitol Today.

[B6] Dunyo SK, Nkrumah FK, Simonsen PE (2000). Single-dose treatment of Wuchereria bancrofti infections with ivermectin and albendazole alone or in combination: evaluation of the potential for control at 12 months after treatment. Trans R Soc Trop Med Hyg.

[B7] Kyelem D, Sanou S, Boatin B, Medlock J, Coulibaly S, Molyneux DH (2003). Impact of long-term ivermectin (Mectizan) on Wuchereria bancrofti and Mansonella perstans infections in Burkina Faso: strategic and policy implications. Ann Trop Med Parasitol.

[B8] Hopkins DR, Eigege A, Miri ES, Gontor I, Ogah G, Umaru J, Gwomkudu CC, Mathai W, Jinadu MY, Amadiegwu S, Oyenekan OK, Korve K, Richards FO (2002). Lymphatic filariasis elimination and schistosomiasis control in combination with onchocerciasis control in Nigeria. Am J Trop Med Hyg.

[B9] Richards FO, Pam DD, Kal A, Gerlong GY, Onyeka J, Sambo Y, Danboyi J, Ibrahim B, Terranella A, Kumbak D, Dakul A, Lenhart A, Rakers L, Umaru J, Amadiegwu S, Withers PC, Mafuyai H, Jinadu MY, Miri ES, Eigege A (2005). Significant decrease in the prevalence of Wuchereria bancrofti infection in anopheline mosquitoes following the addition of albendazole to annual, ivermectin-based, mass treatments in Nigeria. Ann Trop Med Parasitol.

[B10] Weil GJ, Lammie PJ, Weiss N (1997). The ICT Filariasis Test: A rapid-format antigen test for diagnosis of bancroftian filariasis. Parasitology Today.

[B11] Eigege A, Richards F, Blaney D, Miri E, Umaru J, Jinadu M, Mathai W, Hopkins D (2003). Rapid assessment for lymphatic filariasis in central Nigeria: a comparison of the immunochromatographic card test and hydrocele rates in an area of high endemicity. Am J Trop Med Hyg.

